# In Vitro Findings of Titanium Functionalized with Estradiol via Polydopamine Adlayer

**DOI:** 10.3390/jfb8040045

**Published:** 2017-09-28

**Authors:** Chris Steffi, Zhilong Shi, Chee Hoe Kong, Wilson Wang

**Affiliations:** Department of Orthopaedic Surgery, Yong Loo Lin School of Medicine, National University of Singapore, NUHS Tower Block, Level 11, 1E Kent Ridge Road, 119228 Singapore, Singapore; chrissteffi@u.nus.edu (C.S.); dossz@nus.edu.sg (Z.S.); e0012328@u.nus.edu (C.H.K.)

**Keywords:** osteoporosis, implants, estradiol, polydopamine, osteoblasts and osteoclasts

## Abstract

To improve orthopedic implant fixation and reduce post-operative complications, osteogenic molecules are delivered locally by immobilizing them on the surface of implants, which will modulate the biology of cell attachment and differentiation on the implant surface. Estradiol, a natural steroid hormone, maintains bone metabolism by decreasing bone resorption. It either directly or indirectly affects osteoclasts. In this work, estradiol was immobilized on a titanium surface by polydopamine adlayer. Immobilization of estradiol was confirmed by X-ray electron spectroscopy (XPS), immunofluorescence staining and enzyme-linked immunosorbent assay (ELISA). Estradiol-modified substrates enhanced alkaline phosphatases activity (ALP) and calcium deposition of osteoblasts. However, these substrates did not decrease tartrate-resistant acid phosphatase (TRAP) activity and actin ring formation of the osteoclast. The scanning electron microscopic (SEM) images of estradiol-modified substrates showed the formation of estradiol crystals, which decreased the potency of immobilized estradiol. Despite having a successful immobilization of estradiol via the polydopamine technique, the bioavailability and potency of coated estradiol is reduced due to crystallization, suggesting that this is not a suitable system for localized estradiol delivery as tested in vitro here. Consequently, other suitable platforms have to be explored for immobilizing estradiol that will prevent crystal formation while preserving the biological activity.

## 1. Introduction

Estrogen, a female sex hormone, is a key player in bone metabolism. Decline in estrogen production by ovaries in post-menopausal women triggers acceleration of osteoporosis [1]. It is reported that the risk of hip fractures is reduced by 60% if bioavailable estrogen is greater than 5–9 pg/mL [2]. Moreover, administration of exogenous estrogen to post-menopausal women decreased osteoporotic hip fractures by 33% [3]. At cellular level, estradiol inhibits osteoclast differentiation either directly inhibiting osteoclast activity [4] or indirectly, by modulating the gene expression of osteoblasts, T and B cells [5,6]. Systemic estradiol administration has adverse effects such as pulmonary embolism, coronary heart disease, deep vein thrombosis, and breast cancer. Not only does administrating low estradiol doses locally to treat vaginal atrophy have similar beneficial effects as systemic estradiol administration, it also alleviates the adverse reactions [7]. Although local delivery systems are used to treat vaginal atrophy, no system is approved clinically for local estradiol administration to bone. However, research has been carried out to locally deliver osteogenic molecules at bone-implant interface to improve osseointegration.

Modification of implant surfaces with osteogenic molecules is a promising way to control cellular behavior at bone-implant interface and regulate osseointegration. For instance, external fixator pins coated with hydroxyapatite are used to treat osteoporotic fracture of wrists and demonstrated good grip of bone as compared to that of uncoated fixator pins [8]. Anti-osteoporotic drugs such as bisphosphonate were engraved on metal implants. Bisphosphonate-immobilized implants in rat tibiae had 28% higher pullout force than the uncoated controls [9]. However, bisphosphonates are known to cause microfractures in bone [10] and, therefore, surface modification of implants with other desirable osteogenic molecules should be investigated. Few studies are done to release estradiol at the bone-implant interface. It has been reported that the surface modification of titanium with polyelectrolyte assembly estradiol-loaded mesoporous silica nanoparticles enhanced osteoblast differentiation [11]. Nonetheless, silica nanoparticles accelerated the production of reactive oxygen species (ROS), which induced DNA damage [12]. Hence, it may not be an appropriate system for osteoporotic bone.

Polydopamine adlayer formation is a facile technology for surface modifications [13]. The coatings can be achieved by soaking the substrates in polydopamine solution under alkaline conditions. The multiple thiol moieties of polydopamine adlayer are known to bind to thiols and amine functional groups of organic molecules, proteins and peptides. Moreover, non-covalent interactions of polydopamine adlayer such as hydrogen bonding, π–π interactions were also reported [14]. Hence, the non-toxic coatings of polydopamine provide a versatile adlayer for secondary modifications. Growth factors, such as bone morphogenetic protein 2 (BMP-2) and vascular endothelial growth factor (VEGF), as well as polyphenol, like curcumin, have been immobilized on metal implants by polydopamine adlayers [15,16,17], but to our knowledge, no work was done for estradiol. In this study, we explored the possibility of 17β-estradiol (E2, most potent type of natural estrogen) immobilization on titanium (Ti) via polydopamine (pDOP) chemistry. We hypothesized that the pDOP-based surface immobilization of estradiol will preserve the biological activity on the surface, which will be beneficial for augmenting osteoblast differentiation while inhibiting osteoclast function on the implant surface. The modification was characterized by X-ray electron spectroscopy (XPS), immunofluorescence staining, enzyme-linked immunosorbent assay (ELISA), and scanning electron microscope (SEM). Thereafter, osteoblast and osteoclast were cultured on the substrates to study the effects of immobilized E2.

## 2. Results

### 2.1. Surface Characterization

**Figure 1:**
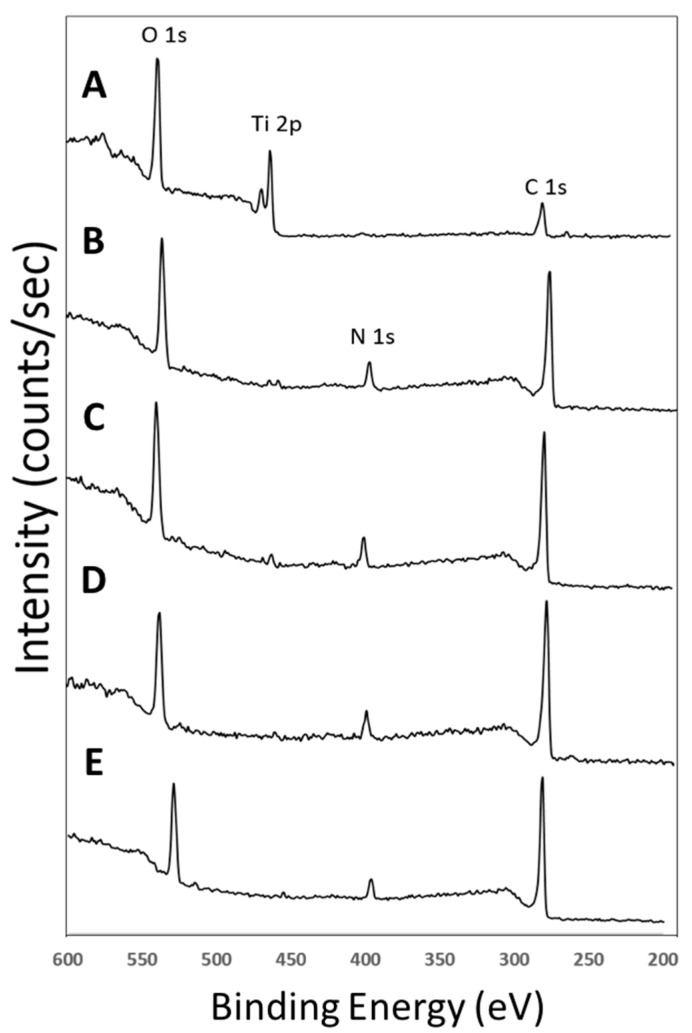
XPS wide-scan spectra of substrates portray E2 immobilization on the surface. (**A**) Ti; (**B**) Ti-pDOP; (**C**) Ti-pDOP-E2-0.1 µg; (**D**) Ti-pDOP-E2-1 µg and (**E**) Ti-pDOP-E2-10 µg. Ti (**A**) displays signals for O 1s, Ti 2p and C1s, (**B**) Ti-pDOP depicts an additional peak of N 1s peak, whereas after estradiol immobilization (**C**–**E**) the signals for N 1s were reduced in a dose dependent manner depicting immobilization of E2 on the surface.

Ti was first coated with pDOP (Ti-pDOP). Thereafter, surface of Ti-pDOP was functionalized separately with three different concentrations of E2 which are designated as Ti-pDOP-E2-10 µg, Ti-pDOP-E2-1 µg, and Ti-pDOP-E2-0.1 µg. Elemental compositions of the substrate surface at different stages of modification were analyzed by XPS. Figure 1 portrayed the XPS wide spectra scan of Ti, Ti-pDOP, Ti-pDOP-E2-0.1 µg, Ti-pDOP-E2-1 µg and Ti-pDOP-E2-10 µg. Wide spectra scan of pristine Ti depicted signal peaks at Ti 2p (460 eV) and O 1s (530 eV) (Figure 1A). In Ti-pDOP substrates, nitrogen and carbon content increased, although there was a decrease in Ti content (Figure 1B, Table 1). After E2 immobilization on Ti-pDOP-E2-0.1 µg, Ti-pDOP-E2-1 µg and Ti-pDOP-E2-10 µg, the nitrogen content decreased in a dose dependent manner (Figure 1C–E, Table 1).

**Figure 2:**
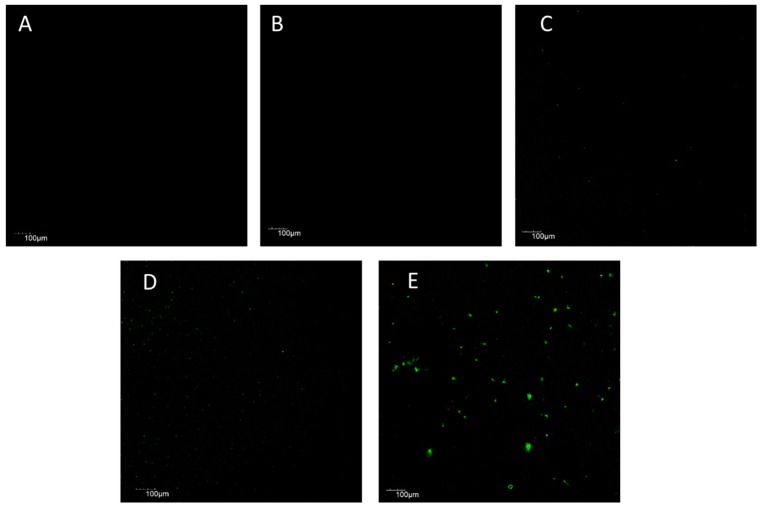
Fluorescence images of substrates before and after modifying with E2. The images of the substrates (**A**) Ti; (**B**) Ti-pDOP; (**C**) Ti-pDOP-E2-0.1 µg; (**D**) Ti-pDOP-E2-1 µg and (**E**) Ti-pDOP-E2-10 µg were obtained by confocal laser scanning microscope (CLSM) after staining for the immobilized estradiol with anti-rabbit IgG–FITC. Green fluorescence was observed on (**C**) Ti-pDOP-E2-0.1 µg; (**D**) Ti-pDOP-E2-1 µg and (**E**) Ti-pDOP-E2-10 µg as compared to controls (**A**) Ti; (**B**) Ti-pDOP. The fluorescence intensity increased with increasing concentrations of immobilized estradiol.

Furthermore, we performed immunofluorescence staining to confirm the presence of E2. The substrates were blotted with anti-estradiol primary antibody and FITC-labeled secondary antibody. Pristine titanium showed no fluorescence (Figure 2A) while polydopamine can bind covalently to nitrogen moieties present in the antibodies. Therefore, the nonspecific fluorescence from polydopamine coated titanium substrates were nullified using software application (Figure 2B) and were used as the baseline to image Ti-pDOP-E2-0.1 µg, Ti-pDOP-E2-1 µg, and Ti-pDOP-E2-10 µg substrates. E2 coated substrates emitted green fluorescence. The fluorescence intensities increased with elevated E2 density immobilized on the substrates. (Figure 2C–E). Large aggregates of estradiol were observed on Ti-pDOP-E2-10 µg (Figure 2E).

**Figure 3:**
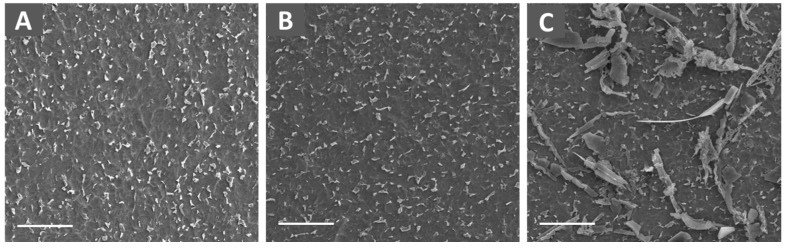
SEM images of substrates before and after the modification with E2. (**A**) Ti; (**B**) Ti-pDOP, (**C**) Ti-pDOP-E2-10 µg (Scale bar = 20 µm). Large crystals of estradiol were observed on (**C**) Ti-pDOP-E2-10 µg.

The amount of immobilized E2 was also quantified by ELISA kit (Table 2). The amounts of bound E2 on the surface of Ti-pDOP-E2-0.1 µg, Ti-pDOP-E2-1 µg and Ti-pDOP-E2-10 µg were tabulated as 0.054 ± 0.1, 0.72 ± 0.11, and 8.15 ± 0.49 μg/cm^2^ respectively (Table 2). Substrates were observed under scanning electron microscope (SEM). As shown in Figure 3C, large needle-shaped crystals and crystals clusters were observed in the E2 modified substrates (Figure 3C) while no such crystals could be found on Ti and Ti-pDOP (Figure 3A,B).

### 2.2. E2 Release

**Figure 4:**
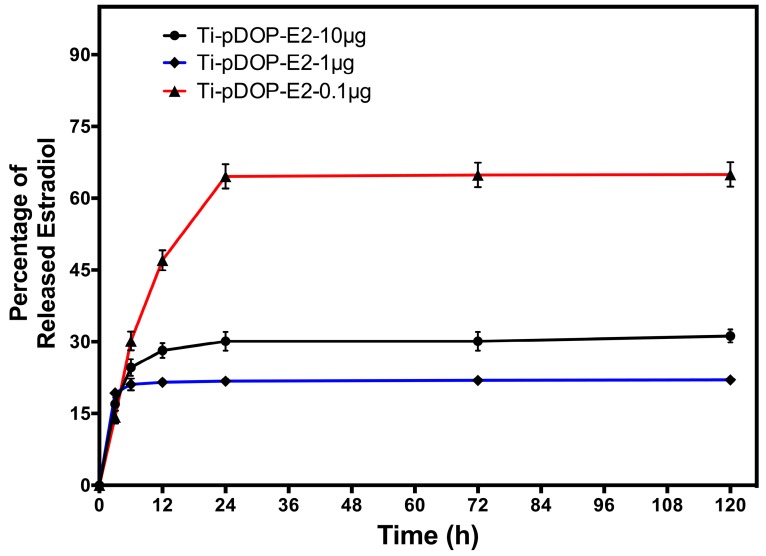
The E2 release profile of E2-immobilized substrates. E2 release was studied using ELISA after incubating E2-immobilized substrates in PBS at 37 °C. After 24 h, 64% of estradiol was released from Ti-pDOP-E2-0.1 µg, 22% of E2 was released from Ti-pDOP-E2-1 µg and 31% from Ti-pDOP-E2-10 µg.

The E2-immobilized substrates were submerged in phosphate buffer saline (PBS) for 5 days and the release profile of bound E2 was studied using ELISA. For Ti-pDOP-E2-1 µg and Ti-pDOP-E2-10 µg, burst release was observed for the initial 6 h, while the burst release in Ti-pDOP-E2-0.1 µg extended up to 24 h (Figure 4). After 24 h in PBS, E2 release from the substrates was below detectable limits (25 pg/mL). Following 5 days of incubation, about 22% of E2 was released from Ti-pDOP-E2-1 µg and 31% from Ti-pDOP-E2-10 µg, but almost 64% was released from Ti-pDOP-E2-0.1ug (Figure 4). The minimal estradiol release after 24 h and crystallization of the bound estradiol (Figure 3C) reduced the bioavailable concentrations of estradiol hence reducing the potency of estradiol. The in vitro effects of estradiol-functionalized substrates on MC3T3-E1 and RAW 264.7 cell line were investigated.

### 2.3. Cell Cytotoxicity Assay

**Figure 5:**
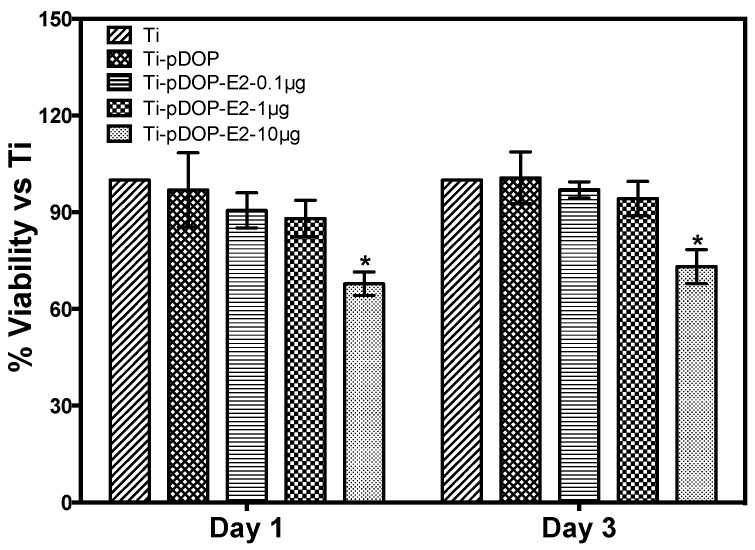
Cell viability and proliferation of MC3T3-E1 cells on substrates before and after the modification with E2. MTT assay was performed at day 1 and day 3 of MC3T3-E1 cell culture on different substrates. Cell proliferation was reduced for the cells cultured on Ti-pDOP-E2-10 µg as compared to Ti (*p* < 0.05)

The cytotoxic effects of different substrates on MC3T3-E1 preosteoblast cells were investigated by MTT assay after day 1 and day 3 of culture (Figure 5). Cell proliferation on Ti-pDOP-E2-0.1 µg and Ti-pDOP-E2-1 µg were similar to Ti and Ti-pDOP (Figure 5) on both days. However, the cell metabolism was significantly reduced for Ti-pDOP-E2-10 µg (Figure 5) as compared to that of pristine Ti.

### 2.4. Osteoblast Differentiation and Calcium Deposition

**Figure 6:**
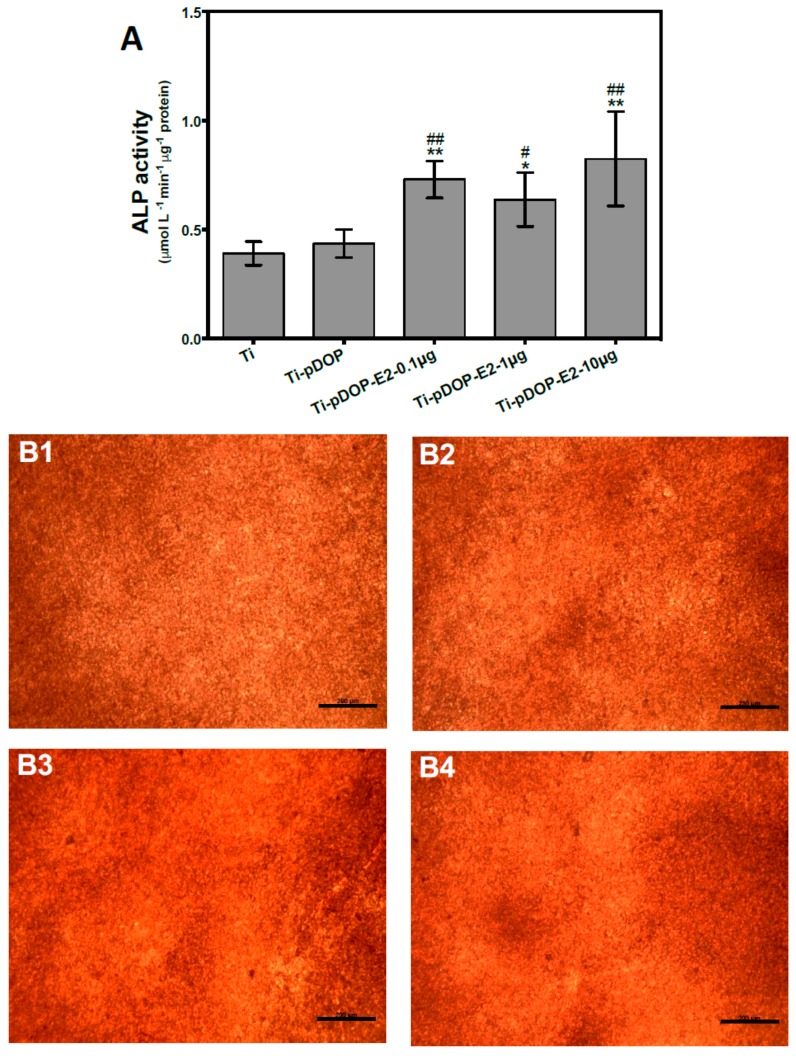

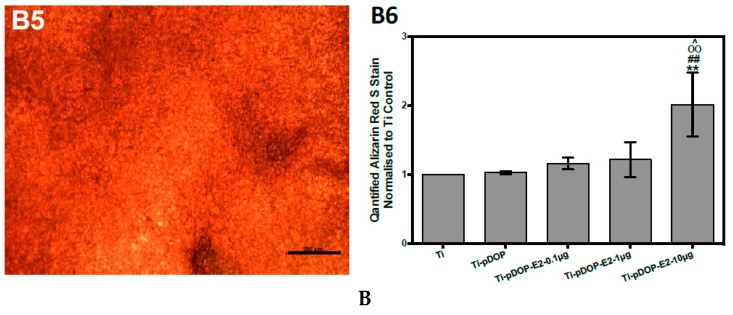
ALP activity (**A**) and calcium deposition (**B**) of osteoblasts on the substrates before and after the modification with E2. (**A**) ALP activity of MC3T3-E1 cells after 1 week culture on different substrates. Estradiol immobilized substrates such as Ti-pDOP-E2-0.1 µg, Ti-pDOP-E2-1 µg and Ti-pDOP-E2-10 µg augmented ALP activity and statistical difference is denoted as (*) *p* < 0.05 versus Ti and (#) is *p* < 0.05 versus Ti-pDOP. (**) and (##) denotes *p* < 0.01. (**B**) Alizarin red S staining after 4-week culture of MC3T3-E1 cells on (**B1**) Ti, (**B2**) Ti-pDOP, (**B3**) Ti-pDOP-E2-0.1 µg, (**B4**) Ti-pDOP-E2-1 µg and (**B5**) Ti-pDOP-E2-10 µg. Scale bar = 200 µm. (**B6**) Quantification of alizarin red S stain. Higher alizarin red S stain was observed on Ti-pDOP-E2-10 µg and the statistical significant difference is represented as (**) as compared to Ti (*p* < 0.01), (##) as compared to Ti-pDOP (*p* < 0.01), (oo) as compared to Ti-pDOP-E2-0.1 µg (*p* < 0.01) and (^) as compared to Ti-pDOP-E2-1 µg (*p* < 0.05).

MC3T3-E1 preosteoblasts were seeded on the different substrates and maintained for 1 week. As shown in Figure 6A, cells cultured on E2 immobilized substrates Ti-pDOP-E2-0.1 µg, Ti-pDOP-E2-1 µg and Ti-pDOP-E2-10 µg showed higher alkaline phosphatase (ALP) activity than that of the pristine Ti and Ti-pDOP (*p* < 0.05). Calcium deposition was studied at the end of 4 weeks via alizarin red S staining (Figure 6(B1–B6)). Calcium deposition in Ti-pDOP-E2-10 µg (Figure 6(B5)) was slightly higher as compared to that of the pristine titanium and Ti-pDOP (Figure 6(B1,B2)). However, substrates with lower E2 densities (Ti-pDOP-E2-0.1 µg and Ti-pDOP-E2-1 µg, Figure 6(B3,B4) had similar calcium deposition pattern to that of pristine Ti and Ti-pDOP (Figure 6(B1,B2)). The irregular staining patterns on the substrates is because of the different thickness of deposited calcium. To verify the results alizarin stain were quantified (Figure 6(B6)). Alizarin red S stain on Ti-pDOP-E2-10 µg were significantly higher than Ti, Ti-pDOP, Ti-pDOP-E2-0.1 and Ti-pDOP-E2-1 µg.

### 2.5. Total DNA of Osteoclast

**Figure 7:**
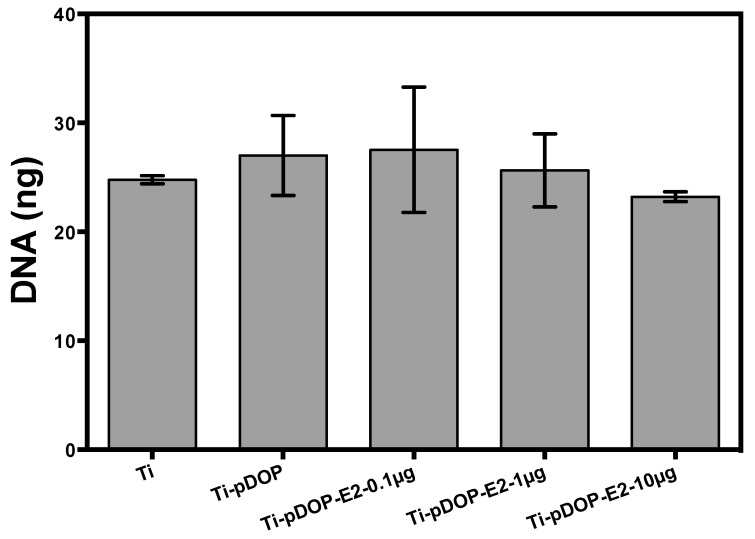
Total DNA of osteoclast on the substrates before and after surface functionalization with E2. After 5 days of culture, total DNA of osteoclast was measured in the cell lysates. Total DNA were similar on all the substrates suggesting that estradiol immobilized substrates did not decrease osteoclast DNA.

RAW 264.7 cells were supplemented with receptor activator of Receptor Activator of NF-κB ligand (RANKL) to induce osteoclast differentiation. After 5 days, total DNA of the cells was measured (Figure 7). The amount of total DNA was similar in all the substrates. No reduction of osteoclast DNA was observed in E2-modified substrates.

### 2.6. Osteoclast Actin Ring Formation and TRAP Activity

**Figure 8:**
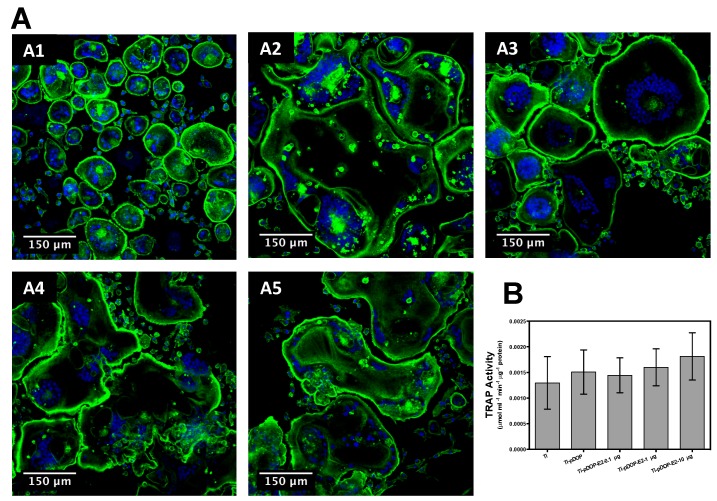
Osteoclast morphology (**A**) and TRAP activity of osteoclasts (**B**) on the substrates before and after the modification with E2. Confocal laser scanning microscope images of osteoclasts, stained for nucleus (blue) and actin (green). Images were acquired after 5 days of osteoclast culture on (**A1**) Ti; (**A2**) Ti-pDOP; (**A3**) Ti-pDOP-E2-0.1 µg; (**A4**) Ti-pDOP-E2-1 µg and (**A5**) Ti-pDOP-E2-10 µg. Multinucleated osteoclast formation with discrete actin rings were observed on all the substrates. (**B**) TRAP activity of osteoclasts after 5 days of culture on the substrates. The TRAP activities of osteoclasts were similar on all the substrates and no reduction was observed on estradiol-modified substrates.

The effects of immobilized E2 were investigated on the differentiation of RAW 264.7 cells under stimulation of RANKL, by investigating the actin ring formation and TRAP activity. TRAP activity, estimated after 5 days of culture (Figure 8B), was not significantly different to that of the Ti and Ti-pDOP. Osteoclasts were stained for actin rings after 5 days of culture on the substrates (Figure 8(A1–A5)). All the substrates presented with multinucleated cells with distinct actin rings. Actin rings of osteoclasts on Ti (Figure 8(A1)) were smaller as compared to the ones on Ti-pDOP (Figure 8(A2), Ti-pDOP-E2-0.1 µg (Figure 8(A3)), Ti-pDOP-E2-1 µg (Figure 8(A4)), and Ti-pDOP-E2-10 µg (Figure 8(A5)).

## 3. Discussion

In this study, functionalization of Ti with E2 via polydopamine adlayer was conducted. The surface modification was examined by XPS analysis. The XPS wide spectra of Ti substrates showed peaks for Ti 2p, O 1s and C 1s. Signals for carbon was due to unavoidable hydrocarbon contamination (Figure 1A), which is conventional in XPS scans [18]. Nitrogen atoms present in dopamine increased the nitrogen content after forming the polydopamine adlayer [16], while titanium content decreased significantly (Figure 1B and Table 1). This confirmed the successful coating of dopamine on the titanium surface. After E2 immobilization on Ti-pDOP adlayer, there was a decline in nitrogen percentage in a dose dependent manner (Figure 1C–E and Table 1), because E2 lacks nitrogen molecule. Decrease in nitrogen percentage indicated a successful immobilization of E2 on polydopamine surfaces. When labeled with anti-E2 antibody, Ti-pDOP-E2-0.1, Ti-pDOP-E2-1 and Ti-pDOP-E2-10 µg emitted fluorescence (Figure 2C–E), and the highest fluorescence intensity was observed in Ti-pDOP-E2-10 µg (Figure 2E). The results of XPS and immunofluorescence staining indicated that E2 had been successfully immobilized on substrates. Furthermore, the amount of immobilized E2 was quantified using ELISA. Increasing the initial loading concentration linearly increased the loading efficiency of bound E2 (Table 2). For instance, the loading efficiency of E2 on Ti-pDOP-E2-0.1 µg was 54%, 72% in Ti-pDOP-E2-1 µg, and further improved to 81.5% in Ti-pDOP-E2-10 µg. This is considerably higher than the loading efficiency (60%) of curcumin achieved previously by our group [16]. As such, E2 had, in fact, been loaded onto the surface at a higher surface density than that of curcumin. The mechanism of binding E2 to polydopamine remains unknown. It is known that polydopamine has non-covalent interactions such as π–π interactions, hydrogen-bonding (H-bonding), ionic and cation-π interactions [14]. H-bonding interactions are well known for hydroxyl groups of E2 [19]. Thus, E2 may form complexes with polydopamine via hydrogen bonding interactions. Further studies are required to validate this phenomenon.

To achieve a therapeutic dose, drug delivery systems are supersaturated with high drug concentration. However, supersaturated concentrations of drugs are thermodynamically unstable, leading to crystal formation [20], which reduces the bioavailable concentrations of drugs. E2 is known to form crystals at higher concentrations and impurities are another factor causing E2 crystallization [21]. E2 coated substrates were observed under SEM to inspect the possibility of crystal formation. As shown in Figure 3C, large needle-shaped crystals and crystal clusters were observed in the E2-modified substrates while no such crystals could be found on Ti and Ti-pDOP (Figure 3A,B). Drug loaded at higher concentration especially in case of steroids such as estradiol induce crystal formations [22]. The crystallization may be because of formation of aggregates at higher concentration. Lowering the concentration may reduce the crystal formation. Nonetheless with a pDOP-based estradiol delivery system used in this study, severe burst release was observed at lower E2 concentrations, which would also reduce the bioavailable concentrations of E2. Even though the formation of crystals is not desirable, we proceeded with the E2 release study and cell study to monitor the potency of immobilized E2.

Burst release of E2 from all the substrates was observed for the initial 6 to 12 h. No measurable E2 was released after 24 h. Even after 5 days in PBS, 69% of E2 remained on Ti-pDOP-E2-10 µg and 78% on Ti-pDOP-E2-1 µg. Though, only 36% of E2 remained on Ti-pDOP-E2-0.1 µg (Figure 4). This indicated that the release profile of E2 was dependent on the surface density of immobilized E2. Results suggested that E2 bound strongly to polydopamine adlayer via mechanisms as discussed earlier.

In vitro cell models for osteoblasts and osteoclasts, which have pivotal roles in bone remodeling, were investigated for the potency of immobilized E2. MC3T3-E1 cells, a mouse preosteoblast cell line, are used widely as an in vitro osteoblast model to study cell-biomaterial interactions. They provide homogeneous cell population with similar growth rate and gene expression profile as human primary osteoblasts [23]. MC3T3-E1 cells are capable of portraying distinct stages during osteoblast development such as proliferation, differentiation, and matrix mineralization. This corresponds with in vivo bone deposition [24]. Additionally, MC3T3-E1 cell line expresses both E2 receptors (ER) α and β [25,26], making it a suitable osteoblast model for this study. It has been reported that cell culture media, supplemented with E2, enhanced proliferation of MC3T3-E1 cells [27]. Another study treated MC3T3-E1 cells to different concentrations of E2 (10^−10^, 10^−9^, 10^−8^ and 10^−7^ mol L^−1^). Cells cultured in 10^−9^, 10^−8^ and 10^−7^ mol L^−1^ of E2 showed higher cell viability than that of untreated controls, and cells treated with 10^−8^ mol L^−1^ E2 exhibited maximum cell viability among all the E2 treated groups [28]. MC3T3-E1 cells display sequential differentiation patterns by expressing ALP in the early phases of differentiation [24,29] while depositing calcium that marks the final phase of differentiation [24]. Osteoblasts treated with E2 in solution enhanced ALP activity and calcium deposition [30,31]. However, the effect of surface immobilized E2 on osteoblast has not been previously investigated. Here, we explored the effect of immobilized E2 on cell viability by MTT assay (Figure 5). Unlike previous reports, the cell viability of MC3T3-E1 cells cultured on Ti-pDOP-E2-0.1 µg and Ti-pDOP-E2-1 µg was similar to the cells cultured on pristine Ti. These contrasting results may be due to the crystallization of E2 on the Ti surface, reducing the bioavailable E2. Intriguingly, a 30% decrease in cell metabolism was noted in the cells cultured on Ti-pDOP-E2-10 µg with high E2 surface density, as compared to that of the pristine Ti (Figure 5). Previous reports suggested that a decrease in osteoblast proliferation was associated with an increase in differentiation markers like ALP and calcium deposition [32]. Therefore, this association can be extrapolated onto the results observed for Ti-pDOP-E2-10 µg (Figure 5). We studied the expression of ALP and calcium deposition on E2-functionalized Ti substrates (Figure 6). As shown in Figure 6A, ALP expression was notably higher in all the E2-immobilized substrates as compared to those of the controls, Ti and Ti-pDOP. The calcium deposition was studied using Alizarin S staining because of its ability to bind to deposited calcium [33]. Calcium deposition was higher in osteoblasts cultured on Ti-pDOP-E2-10 µg (Figure 6(B5,B6)) as compared to the controls (Figure 6(B1,B2,B6)), while no increase in mineralization was observed in Ti-pDOP-E2-0.1 µg and Ti-pDOP-E2-1 µg (Figure 6(B3,B4,B6)). Although previous studies reported the effects of E2 in solution on osteoblast functions [30,31], our study is the first to explore the outcomes of surface immobilized E2 for osteoblast differentiation markers. On Ti-pDOP-E2-10 µg substrates, both early differentiation marker ALP and late differentiation marker calcium deposition of osteoblasts increased (Figure 6) whereas osteoblast proliferation decreased (Figure 5). These results indicated that the Ti-pDOP-E2-10 µg stimulated osteoblast differentiation over proliferation. Varied responses of osteoblast to different E2 surface densities suggested that osteoblast development on implant surface can be further tailored and a more detailed research has to be carried out to elucidate the molecular pathways involved.

The outcome of immobilized E2 on bone resorbing osteoclast was also investigated in this study. RANKL treatment can stimulate osteoclast differentiation in RAW 264.7 cells [34]. Easy access, simple culture techniques, and pure population of macrophage/pre-osteoclast obtained from RAW 264.7 mouse macrophage cell line make it an attractive in vitro model [35]. Research had already shown that E2 can induce apoptosis in osteoclasts by Fas/FasL signaling [4,36], thereby decreasing osteoclast cell number. Cell proliferation and cell number has been estimated previously, by measuring DNA quantity of cells [37,38]. The total DNA of osteoclasts was estimated to assess the ability of E2-modified substrates on the osteoclast proliferation. The DNA of osteoclast cultured on E2-modified substrates was statistically similar to the controls Ti and Ti-pDOP (Figure 7). The surface modification with E2 did not inhibit the proliferation of osteoclasts. Furthermore, we explored the effects of immobilized E2 on osteoclast differentiation markers such as TRAP activity, actin ring formation and multinucleated cell formation. Previous studies reported that media supplemented with E2 inhibited the RANKL induced osteoclast differentiation of primary cells from mouse bone marrow and pre-osteoclast cell line RAW 264.7 [39]. E2 was also reported to inhibit RANKL-induced osteoclast differentiation of human monocytes [40]. Active osteoclasts secrete TRAP and the amount of TRAP correlates with osteoclast activity and osteoclast cell number [41,42]. This makes TRAP an attractive marker for bone resorption [43]. Apart from TRAP expression, osteoclast differentiation of RAW 267.4 cells leads to rearrangement of actin cytoskeleton, cell-cell fusion, formation of acting ring and ruffled borders to produce multinucleated bone-resorbing cells [44]. Therefore, multinucleated actin ring formation indicates osteoclast cell formation. In the present study, multinucleated osteoclast formation and TRAP activity was monitored to study osteoclast differentiation of RAW 264.7 on E2-modified and unmodified Ti substrates. Multinucleated osteoclast was observed in all the substrates (Figure 8(A1–A5)) and no disruption of actin ring was observed in E2-modified substrates (Figure 8(A3–A5)). Moreover, in contrast to the previous reports [45,46] no decrease in TRAP activity was observed on E2-modified substrates (Figure 8B). It has been reported that E2 and RANKL regulation of osteoclasts is dependent on TNF receptor-associated factor 6 (Traf6) for downstream signaling [40]. In the presence of E2, estradiol-estrogen receptor-alpha (ERα) complex sequesters Traf6, making it unavailable for RANK-RANKL signaling, which inhibits osteoclastogenesis. Despite of supersaturated concentrations of E2 on Ti surface, crystallization and decline in E2 release from the surface after 24 h may significantly reduce the bioavailable concentrations of E2, causing Traf6 to be available for RANK-RANKL signaling for osteoclast differentiation of RAW 264.7. Thus, osteoclast differentiation is not inhibited.

## 4. Materials and Methods

### 4.1. Materials

Titanium/Aluminum/Vanadium Alloy (Ti-6Al-4V, denoted as Ti) substrates were obtained from Goodfellow Cambridge Ltd. (Huntingdon, UK). Mouse cell line RAW 264.7 of macrophage lineage and murine osteoblast cell line MC3T3-E1 subclone 14 were purchased from the American Type Culture Collection (Manassas, VA, USA). All chemicals and reagents were obtained from Sigma-Aldrich (St. Louis, MO, USA) unless otherwise stated and used as received. Milli-Q water (>18.2 MΩ cm; Arium 611UF, Sartorius Stedim Biotech, Göttingen, Germany) was used in the experiments.

### 4.2. Substrate Preparation

Ti foils (1 × 1 cm^2^) were polished using 1200-grid sandpaper and were sonicated in water for 10 min. The foils were cleaned by ultrasonicating in Kroll’s reagent (7.2% HNO_3_, 4.0% HF, 88.8% water) for 10 min [47]. 1N sodium hydroxide solution was used to terminate the reaction. For surface passivation, the substrates were immersed in 40% nitric acid for 40 min. Then, they were washed with copious amounts water. Washed Ti substrates were coated with polydopamine (Ti-pDOP) as previously described [48]. In brief, Ti substrates were immersed overnight in dopamine solution (2 mg/mL of dopamine hydrochloride in 10 mM of Tris Buffer at pH = 8.5). The reaction was carried out in dark. Subsequently, Ti-pDOP substrates were washed thoroughly in water to clear away unbound dopamine before drying under nitrogen flow.

Various concentrations of E2 loading solutions (10 μg/50 μL, 1 μg/50 μL, 0.1 μg/50 μL in absolute ethanol) were prepared and 50 µL of each concentration was carefully transferred onto the surface of Ti-pDOP substrates. They were then gently rinsed three times with water to wash away unbound E2 after 24 h. The prepared substrates were denoted as Ti-pDOP-E2-10 µg, Ti-pDOP-E2-1 µg, and Ti-pDOP-E2-0.1 µg, respectively.

### 4.3. Characterization

The surface chemical make-up at the different stages of modification was examined by XPS using a Kratos AXIS Ultra DLD spectrometer (Kratos Analytical Ltd., Manchester, UK) with an Al Kα X-ray source (1486.7 eV) [49]. C1s (C–C bond) peak at 284.6 eV was applied as a reference to compute all other binding energies. SEM (JSM-6701F-JEOL, Tokyo, Japan) was used to observe the substrate surfaces. They were first coated with gold by sputter-coating at 10 mA for 50 s. Images were acquired at 5 different regions per substrate at a voltage of 12 kV.

Anti-E2 antibody was used to visualize the bound-E2 on substrates. These substrates were incubated overnight in 3% bovine serum albumin (BSA) at room temperature (RT), before immersing in rabbit anti-E2 antibody for 3 h at RT. Substrates were washed in PBS thrice and incubated with anti-rabbit IgG–FITC for 30 min in dark at RT. They were then washed again in PBS. Finally, these substrates were ready to be observed under confocal laser scanning microscope (CLSM, Olympus FV1000, Tokyo, Japan). The amount of immobilized E2 was indirectly quantified by calculating the unbound E2 washed out in water. Unbound E2 was quantified using ELISA kit for E2 (DRG International, Inc., Springfield, NJ, USA) as per manufacturer’s instructions.

### 4.4. E2 Release Assay

The respective release of E2 from Ti-pDOP-E2-10 µg, Ti-pDOP-E2-1 µg, and Ti-pDOP-E2-0.1 µg were measured at various time points (3, 6, 12, 24, 72, and 120 h). The substrates were immersed in PBS at 37 °C and the released E2 was assayed using E2 ELISA kit as described above.

### 4.5. Cell Culture of MC3T3-E1 and RAW 264.7 Cells

Preosteoblasts MC3T3-E1 were maintained in Alpha Minimum Essential Medium (Invitrogen, Waltham, MA, USA) supplemented with 10% fetal bovine serum (FBS), 100 U/mL penicillin, and 100 μg/mL streptomycin (Invitrogen). For osteoblast differentiation studies, cells were seeded at a density of 30,000 cells/cm^2^ and cultured in differentiation media constituting the supplemented Alpha Minimum Essential as above with the addition of 10 mM sodium β-glycerophosphate and 50 μg/mL ascorbic acid.

RAW 264.7 cell culture was cultured in Dulbecco’s Modified Eagle’s Medium (DMEM) with 10% heat inactivated FBS, 100 U/mL penicillin, and 100 μg/mL streptomycin. To stimulate differentiation of RAW 264.7 cells, 30,000 cells/ cm^2^ were cultured in Alpha Minimum Essential Medium (Invitrogen) with the addition of 10% charcoal dextran stripped FBS, 100 U/mL penicillin, 100 μg/mL streptomycin, and 50 ng/mL of RANKL (R&D Systems, Minneapolis, MN, USA). Cells were maintained at 37 °C in a humidified atmosphere with 5% CO_2._ The media were removed and replenished every 3 days. Substrates were UV-sterilized for 30 min before experiments. All the substrates were placed in 24 well plates before carefully loading 0.1 mL of cell suspension on the substrates. After 6 h of incubation, 0.9 mL of fresh media was added.

### 4.6. Cytotoxicity Assay

MC3T3-E1 preosteoblasts were cultured on different substrates. After day 1 and day 3 of culture, the cells were examined for their metabolic activity using MTT reagent (3-[4,5-dimethyl-thiazol-2-yl]-2,5-diphenyltetrazolium bromide) as previously described [50]. Briefly, culture media was removed from the wells. 450 µL of fresh media and 50 µL of MTT solution (5 mg/mL) were added to cells and incubated at 37 °C. After 4 h, the mixture was removed and the formazan complexes were dissolved in DMSO. Absorbance was measured at 570 nm and the values were normalized with the control (cells cultured on pristine Ti).

### 4.7. ALP Activity Assay

MC3T3-E1 cells were cultured on the substrates for 7 days in differentiating medium. ALP activity was quantified using QuantiChrom™ Alkaline Phosphatase Assay Kit (BioAssay Systems, Hayward, CA, USA) as per manufacturer’s instructions. Briefly, cells were rinsed with PBS and lysed in 0.2% Triton X-100. 50 µL of the cell lysate was reacted with 150 µL of kit working solution. P-nitrophenol (pNP) produced has an optical density measured at 405 nm on 0 min and 4 min in a microplate reader (Synergy H1, BioTek Instruments Inc., Winooski, VT, USA). ALP activity was calculated as per formula provided in the assay kit. Total protein in the cell lysate was estimated using Micro BCA Protein Assay Kit (Thermofischer Scientific, Waltham, MA, USA) with known concentrations of bovine serum albumin (BSA) as standards. The ALP activity was normalized to protein content.

### 4.8. Calcium Deposition

After 4 weeks of MC3T3-E1 cell culture on substrates in differentiating medium, cells were stained for deposited calcium. After washing the substrates with cells in PBS thrice, ice cold 70% ethanol was used to fix the cells for 1 h. After the incubation, ethanol was removed and the cells were washed three times with water. Cells were then stained in 2% alizarin red S solution for another hour. Following the thorough wash with water, cells on the substrate were observed under a microscope, Leica DM LM (Leica Microsystems, Wetzlar, Germany). Alizarin red S was quantified as previously described [51]. In brief, the stained cells were immersed 10% acetic acid with gentle shaking. After 30 min of incubation at RT the absorbance was measured at 405 nm using a microplate reader. The values were normalized with control (pristine Ti).

### 4.9. DNA Quantification

RAW 264.7 cells were seeded on different substrates in the presence of RANKL to induce osteoclast differentiation. After 5 days of culture, osteoclasts were washed with PBS. Multiple freeze-thaw cycles were done to the cells in Milli-Q water. Nuclear-binding dye picogreen reagent (Quant-iT Picogreen dsDNA assay kit, Thermofischer Scientific, Waltham, MA, USA) was used to quantify total DNA by mixing cell lysate with the reagent for 5 min. Excitation at 480 nm, and fluorescence emission at 520 nm was recorded with a microplate reader (Synergy H1).

### 4.10. TRAP Activity of Osteoclasts

After five days of culture, osteoclasts were lysed in 0.2% triton X-100 and TRAP activity was estimated using TRAP assay kit (Takara, Shiga, Japan) as per manufacturer’s instructions. In brief, the cell lysate was mixed with assay buffer (1:1) for 60 min at 37 °C. Reaction was terminated with 0.5 N NaOH and the amount of pNP produced was measured at 405 nm using a microplate reader (Synergy H1). Concentrations of pNP were calculated using a pNP standard curve. The TRAP activity was normalized to total protein of cell lysate. TRAP is expressed as µM of pNP produced per min per µg of protein.

### 4.11. Actin Ring Staining

After culturing for 5 days under stimulation of RANKL, RAW 264.7 cells were rinsed in PBS and fixed with 4% paraformaldehyde for 15 min. The cells were then soaked in 0.1% Triton X-100 solution for 5 min to ensure cell permeabilization. Osteoclast actin was stained with phalloidin Alexa fluor 488 (1:40 dilution, Invitrogen) for 30 min. After washing the cells with PBS, the nuclei were counterstained with DAPI. Cells were washed again before being observed under a CLSM (Olympus FV1000). Images were acquired at 5 different spots and cells with three or more nuclei were taken as osteoclasts.

### 4.12. Statistical Analysis

All the experiments were replicated three times and the results are stated as mean ± SD. Data was analyzed using analysis of variance (ANOVA). *p* < 0.05 was reflected as statistically significant.

## 5. Conclusions

In this study, E2 was successfully immobilized on titanium substrates via polydopamine adlayer. Immobilized E2 increased osteoblast differentiation while the proliferation of osteoclasts was unaffected. The immobilized E2 had no effect on RANKL-induced osteoclast differentiation. Inability of the surface-functionalized E2 to impede osteoclast differentiation may be attributed to crystallization of E2 and poor release resulting in attenuated activity. Crystal formations can either be due to high concentrations of drugs [22] or impurities [21]. Usually, crystallization inhibitors [20] and co-solvents [52] are used to prevent drug crystallizations at supersaturated concentrations, which can be considered for further modification of the current approach to polydopamine-based E2 loading. Even though E2 was immobilized with this current technique, there still exists a need to explore other systems that can do so without inducing the crystal formations.

## Figures and Tables

**Table 1:** Elemental Composition * at the surface as determined by XPS.

Substrates	O%	Ti%	N%	C%
Ti	47.89	38.29	1.44	38.29
Ti-pDOP	19.20	0.70	7.27	72.84
Ti-pDOP-E2-0.1 µg	20	0.66	6.06	73.22
Ti-pDOP-E2-1 µg	18.21	0.27	5.83	75.69
Ti-pDOP-E2-10 µg	18.86	0.42	4.65	76.07

* Percentages calculated based on the Ti, O, N, and C contents only.

**Table 2:** Surface density of immobilized E2 on titanium surface.

Substrates	Total Amount of E2 in Loading Solution (μg)	Amount of E2 in the Wash Solution (μg)	Surface Density of Loaded E2 (μg/cm^2^)
Ti-pDOP-E2-0.1 µg	0.1	0.046 ± 0.1	0.054 ± 0.1
Ti-pDOP-E2-1 µg	1	0.28 ± 0.11	0.72 ± 0.11
Ti-pDOP-E2-10 µg	10	1.85 ± 0.49	8.15 ± 0.49
